# A comparative cell wall analysis of *Trichoderma* spp. confirms a conserved polysaccharide scaffold and suggests an important role for chitosan in mycoparasitism

**DOI:** 10.1128/spectrum.03495-23

**Published:** 2024-06-25

**Authors:** Lisa Kappel, Long Yu, Carolina Escobar, Demetrio Marcianò, Vaibhav Srivastava, Vincent Bulone, Sabine Gruber

**Affiliations:** 1 Department of Bioengineering, University of Applied Sciences, Vienna, Austria; 2 Department of Microbiology, University of Innsbruck, Innsbruck, Austria; 3 School of Agriculture, Food and Wine, The University of Adelaide, Adelaide, Australia; 4 Department of Agricultural and Environmental Sciences, via Celoria 2, Milan, Italy; 5 Division of Glycoscience, Department of Chemistry, School of Engineering Sciences in Chemistry, Biotechnology and Health, KTH Royal Institute of Technology, AlbaNova University Centre, Stockholm, Sweden; Universidade de Brasilia, Brasilia, Brazil

**Keywords:** *Trichoderma*, fungal cell wall, mycoparasitism

## Abstract

Fungal cell walls are dynamic extracellular matrices that enable efficient adaptation to changing environments. While the cell wall compositions of yeasts, human, and plant pathogenic fungi have been studied to some extent, the cell walls of mycoparasites remain poorly characterized. *Trichoderma* species comprise a diverse group of soil fungi with different survival strategies and lifestyles. The comparative study of cell wall carbohydrate-active enzymes in 13 *Trichoderma* spp. revealed that the types of enzymes involved in chitin and chitosan metabolism are phylogenetically distant between mycoparasitic and saprotrophic species. Here, we compare the carbohydrate composition and function of the cell wall of a saprotrophic strain *Trichoderma reesei* with that of the mycoparasitic, biological control agent *Trichoderma atroviride*. Monosaccharide and glycosidic linkage analyses as well as dual *in situ* interaction assays showed that the cell wall polysaccharide composition is conserved between both species, except for the amounts of chitin detected. The results suggest that the observed accumulation of chitosan during mycoparasitism may prevent host recognition. Remarkably, *Trichoderma atroviride* undergoes dynamic cell wall adaptations during both vegetative development and mycoparasitism, which appears to be confirmed by an evolutionarily expanded group of specialized enzymes. Overall, our analyses support the notion that habitat specialization is reflected in cell wall architecture and that plastic chitin remodeling may confer an advantage to mycoparasites, ultimately enabling the successful invasion and parasitism of plant pathogens. This information may potentially be exploited for the control of crop diseases using biological agents.

**IMPORTANCE:**

*Trichoderma* species are emerging model fungi for the development of biocontrol agents and are used in industrial biotechnology as efficient enzyme producers. Fungal cell walls are complex structures that differ in carbohydrate, protein, and enzyme composition across taxa. Here, we present a chemical characterization of the cell walls of two *Trichoderma* spp., namely the predominantly saprotrophic *Trichoderma reesei* and the mycoparasite *Trichoderma atroviride*. Chemical profiling revealed that *Trichoderma* spp. remodel their cell wall to adapt to particular lifestyles, with dynamic changes during vegetative development. Importantly, we found that chitosan accumulation during mycoparasitism of a fungal host emerged as a sophisticated strategy underpinning an effective attack. These insights shed light on the molecular mechanisms that allow mycoparasites to overcome host defenses and can be exploited to improve the application of *T. atroviride* in biological pest control. Moreover, our results provide valuable information for targeting the fungal cell wall for therapeutic purposes.

## INTRODUCTION

Fungal cell walls are complex extracellular matrices whose carbohydrate polymer and protein composition and structure change dynamically to adapt to the encountered environment. The composition of the cell wall has been resolved in multiple fungal species, e.g., *Saccharomyces cerevisiae* (1) and human pathogenic fungi (2). In addition, cell wall surfaces are considered a target of predatory fungi, i.e., mycoparasites, which are gaining increased attention as biocontrol agents of plant pathogens in agriculture. Mycoparasitism is considered to be the ancestral lifestyle of *Trichoderma* spp., and several species have been used for the control of plant diseases, such as *Trichoderma harzianum*, *Trichoderma virens,* and *Trichoderma atroviride* (3). Furthermore, saprotrophic organisms such as *Trichoderma reesei* have long been known as industrially important hosts for protein expression and production. Despite the importance of interspecies fungal interactions and their exploitation in biocontrol strategies, little is known about the cell wall structure of mycoparasites. Moreover, the cell wall architecture of *Trichoderma* spp. is not fully characterized and the few studies available have led to contradicting findings [reference (4) and citations therein]. To address this knowledge gap, we aimed to decipher the cell wall composition of *Trichoderma* by comparing two species with different lifestyles, i.e., the mycoparasite *T. atroviride* and the saprotroph *T. reesei*.

The fungal cell wall is typically composed of an outer amorphous layer containing alkali-soluble carbohydrates and glycoproteins (1, 5) and inner scaffolds of fibrous load-bearing polysaccharides, mainly glucans and chitin (2). β-1,3-Glucan, a homopolymer of covalently linked glucosyl units, accounts for 65%−80% of the cell wall in filamentous fungi. Chitin is a relatively minor but vital component of the cell wall with reported amounts in Basidiomycetes and Ascomycetes ranging from 0.5% to 15% of the total cell wall carbohydrates, and up to 40% in Mucoromycetes (1, 6, 7). In certain types of fungi, chitin is further processed by chitin deacetylases (CE4, EC 3.5.1.41), which increases cell wall flexibility, impacts invasion of plant hosts by plant pathogens (8), and might also be relevant for successful mycoparasitism (9).

Structural (glyco)proteins and enzymes involved in cell wall metabolism account for up to 30%−50% of the dry weight of the fungal cell wall in yeast and 20%−30% in the hyphae of filamentous fungi (10). Apart from their role in the synthesis and reorganization of wall components and structural functions, such as cell shape determination, control of adhesion, and adsorption to substrate surfaces, they are involved in signal transmission and protection against environmental stresses (11). Thus, the enzymes and structural proteins within the fungal cell wall play vital roles and are considered interesting targets for the development of new antifungal drugs. The variety of cell wall-related proteins in human pathogenic fungi has been reviewed (12), leading to the identification of 4,014 cell wall proteins in 24 fungal species. The core enzymes responsible for carbohydrate biosynthesis have also been studied to some extent. For instance, the genomes of *Aspergillus* spp. and *Neurospora crassa* both bear a single gene coding for a catalytic enzyme for β-1,3-glucan synthesis [FKS1, glycosyltransferase family 48 (GT48), EC 2.4.1.34], while the genome of *S. cerevisiae* contains three such genes (13, 14). Chitin is generated by chitin synthases as a homopolymer of β-1,4-linked *N*-acetylglucosaminyl (GlcNAc) residues (15). We recently identified eight membrane-associated chitin synthases (GT2, EC 2.4.1.16) in *Trichoderma* spp. (9), which concur with the number of chitin synthases described in other Sordariomycetes. Despite these findings, data on the synthesis of glucans and other polysaccharides in *Trichoderma* are limited. Here, we performed a comparative *in silico* analysis of cell wall-related proteins in 13 mycoparasitic *Trichoderma* species.

Cell wall remodeling occurs continuously throughout all different stages of fungal development, which provides resilience in adverse growth conditions (2). During fungal interactions, secreted compounds from predators and hosts, e.g., toxins and lytic enzymes (16, 17), are permanent stressors. In addition, the chitinous backbone serves as a target of chitinases [CHI, glycoside hydrolase family 18 (GH18), EC 3.2.1] and chitosanases (CHO, GH75, EC 3.2.1.132), which are secreted in high amounts by mycoparasites to facilitate host invasion (9, 18, 19). In addition, chitin deacetylases [CDA, carbohydrate esterase family 4 (CE4), EC 3.5.1.41], chitosanases, and chitinases are key enzymes secreted in high amounts by *Trichoderma* spp. during mycoparasitism (9, 20). But a conclusive evidence of how *Trichoderma* spp. protect themselves from their own and host-derived hydrolytic enzymes has been missing (19). Cell surface proteins, such as hydrophobins, have been suggested to protect the mycoparasitic cell wall of *Trichoderma* spp. from their own enzymes to some extent, but also play a role in development and plant communication (21). The confinement of polysaccharides to subsurface areas in plant pathogens upon contact with mycoparasites was reported as a defense strategy (22). In plant and human pathogenic fungi, the conversion of surface-exposed cell wall chitin into chitosan by chitin deacetylases appears to be a common strategy (23, 24). Chitosan is particularly enriched in most pathogenic and parasitic fungi (8, 25–27). Importantly, how mycoparasites such as *Trichoderma* bypass the host’s defensive reaction during fungal attack and whether chitosan plays a role in mycoparasites are hitherto unknown. Recently, we were able to show that chitin- and chitosan-modifying enzymes are induced upon mycoparasitism, and hypothesized that chitosan might be important for successful invasion of a host (9). To further substantiate these results, we investigated the changes in chitin and chitosan levels in the fungal cell wall during mycoparasitic attack.

Here, we present a detailed *in silico* analysis of the enzymes involved in cell wall assembly in 13 *Trichoderma* spp. and show that expansion of chitin-active enzymes is the major distinction between the three clades of *Trichoderma*. We provide a comprehensive analysis of the polysaccharide composition of the mycelium during different environmental conditions and the asexual conidia of two selected species, *T. atroviride* and *T. reesei*. Furthermore, our cell wall compositional analyses show that the main building blocks of the cell wall are conserved in the *Trichoderma* species studied, except for elevated chitin/chitosan levels in *T. atroviride*, which clearly distinguishes the mycoparasite from *T. reesei*. Consistent with this observation, high chitin synthase activity was detected in cell-free extracts of *T. atroviride*. We further investigated the adaptability of the mycoparasitic cell wall upon host confrontation and confirmed high levels of chitin deacetylation. The chitin and chitosan content increased in the apical zone of hyphal cells of *T. atroviride* during the interaction with the host. Altogether, these findings improve our understanding of the adaptive architecture of cell wall polysaccharides and proteins in *Trichoderma* spp. In addition, the data suggest that chitosan plays a role during mycoparasitic attack and interspecies interactions.

## RESULTS

### A comparative survey of cell wall carbohydrate-active enzymes revealed increased numbers of chitin processing enzymes in mycoparasitic *Trichoderma* species

**Fig 1:**
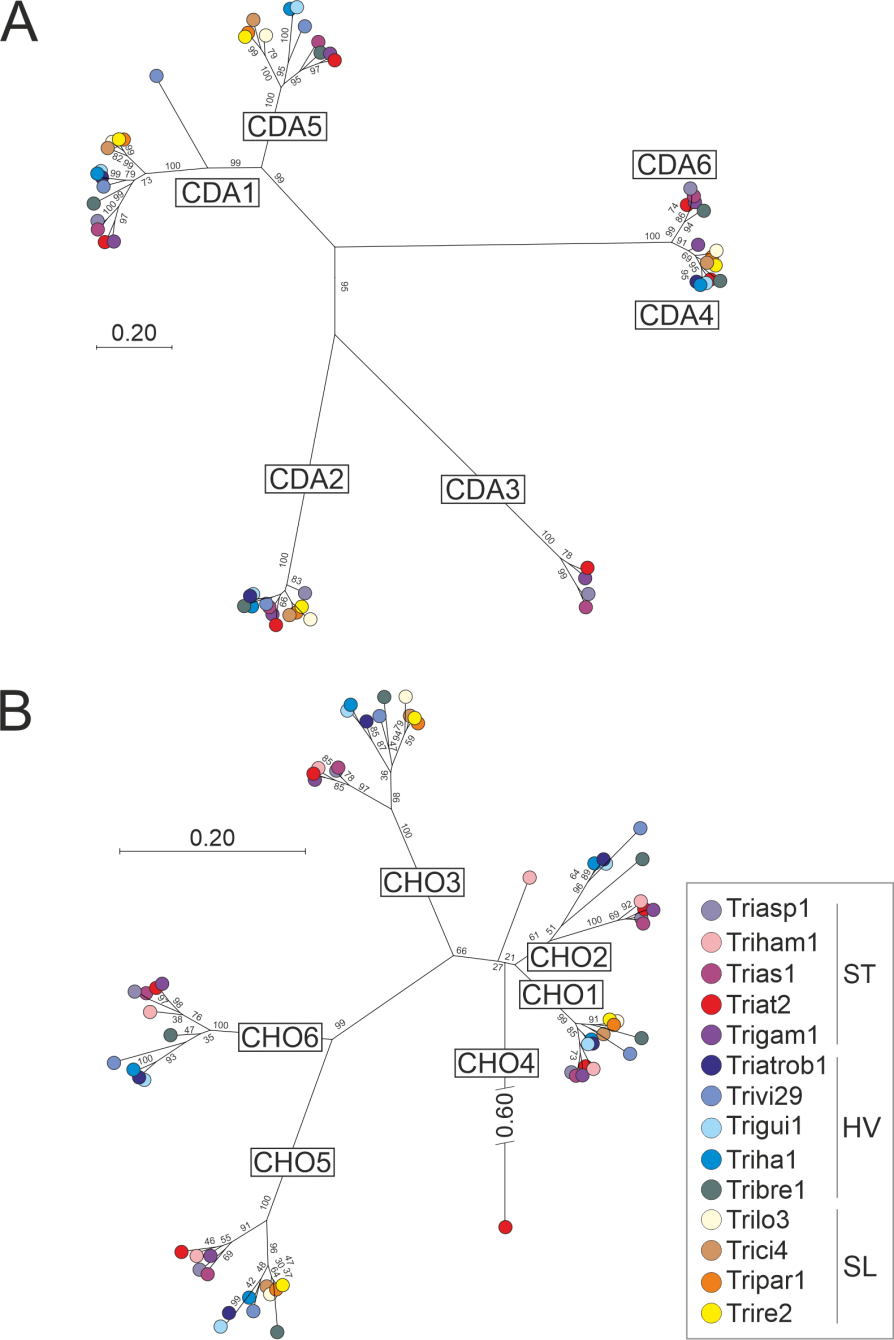
Chitin and chitosan metabolic enzymes are the major distinctions between mycoparasitic and saprotrophic *Trichoderma* spp. Phylogenetic tree of (**A**) CDA and (**B**) CHO from 14 *Trichoderma* spp. Neighbor joining with 500 bootstraps was used and support values are indicated on branches. Nodes are color-coded to represent the species as specified in the box. Evolutionary clades are indicated as mycoparasitic (ST) and (HV), and saprotrophic (SL) clade (see also Table S1).

To gain a concise overview of the enzymes involved in cell wall metabolism in *Trichoderma* spp., we first screened 13 genomes from the well-curated *Trichoderma* genome database (https://mycocosm.jgi.doe.gov). Homologs of cell wall-related proteins of human pathogenic fungi were identified (12). *Trichoderma* spp. are grouped into three evolutionary clades (28): the ancestral mycoparasitic clade “Section Trichoderma” (ST), which gave rise to the two evolutionary younger clades, i.e., the mycoparasitic “Harzianum/Virens” (HV) and the saprophytic “Section Longibrachiatum” (SL). Inferred by homology and prior reports (3, 28), the analysis revealed that between 262 and 316 enzymes are associated with cell wall metabolism in each species (Table 1; Table S2), including the particularly enriched GH, GT, and CE (Tables S2 to S7). Interestingly, the mycoparasitic clades (ST, HV) showed increased numbers of chitin processing enzymes in all species compared to the saprophytic SL clade (on average 12 ± 4 additional enzymes). The clade HV shows a higher variability in chitin processing enzymes than clade ST, possibly indicating the existence of two subgroups within this clade. In particular, GH75 (chitosanases, EC3.2.1.132) and CE4 (chitin deacetylases, EC3.5.1.41) were more abundant and diversified in mycoparasites compared to saprotrophs. As shown by phylogenetic analysis (Fig. 1A and B), only three chitin deacetylases [homologous to *Ta*CDA1, *Ta*CDA2, and *Ta*CDA4 (9)] and three chitosanases [homologous to *Ta*CHO1, *Ta*CHO3, and *Ta*CHO5 (9)] were identified in all species. CHO2 and CHO6 appeared to be specific to mycoparasites and are phylogenetically distant from the CHO1 and CHO5 groups, respectively. A sixth chitosanase (CHO4) is present only in *Trichoderma hamatum* and *T. atroviride*. Interestingly, besides the high homology between CDA1 and CDA5, the other chitin deacetylases are less conserved than the chitosanases (average pairwise distance of 0.682 and 0.385, respectively).

**TABLE 1:** CAZymes involved in cell wall metabolism in *Trichoderma* spp.^*a*^

	Mycoparasitic clade ST				Mycoparasitic clade HV					Saprotroph clade SL			
Component	*Triasp1*	*Trias1*	*Trigam1*	*Triat2*	*Triatrob1*	*Trivi29*	*Trigui1*	*Triha1*	*Tribre1*	*Trici4*	*Tripar1*	*Trire2*	*Trilo3*
Chitin	62	60	61	64	56	68	58	64	69	49	51	50	50
Glucan	87	84	88	70	85	70	92	94	88	76	72	74	71
Glycosylation	58	56	54	49	47	46	54	52	50	52	50	51	49
GPI^*a*^ anchor	10	10	10	9	8	8	10	10	9	9	10	9	10
Mannan	42	41	43	38	38	31	42	43	43	36	35	36	37
Other	56	54	56	48	43	53	53	53	52	48	49	47	45
Total	315	305	312	278	277	276	309	316	311	270	267	267	262

^*a*^ GPI: Glycosylphosphatidylinositol.

As opposed to the enzymes involved in chitin metabolism, families related to glucan metabolism show an inconsistent picture among the mycoparasites (on average 80 ± 9). While the saprotrophic SL clade contains 73 ± 2 glucan-related enzymes (Table 1), some members within the ST and HV mycoparasitic clades show 14 ± 4 additional enzymes. These include *T. harzianum*, *Trichoderma guizhouense*, *Trichoderma asperelloides*, and *Trichoderma gamsii*. In contrast, *T. atroviride* and *T. virens* contain a comparable number of enzymes (70) to the saprotrophic species.

Variations in numbers of enzymes are less pronounced in the remaining enzyme families: between 50 and 55 enzymes have proposed functions in glycosylation and 35–43 enzymes are proposed to function in mannan biosynthesis. Note that in comparison to human pathogenic fungi (12), no homologous enzyme to α-1,3-glucan synthase (GT5, EC 2.4.1.183) nor α-1,3-fucosyltransferase (GT10, EC 2.4.1.152) could be identified *in silico* in neither of the *Trichoderma* spp.

β-1,3-Glucan is the major cell wall component in most fungi. Interestingly, in several species, chitin is covalently linked to glucans to form a basket-like core scaffold, further strengthened by intrachain hydrogen bonds in the glucan and chitin polymers (2). Similar to *Aspergillus* and *Neurospora*, a single β-1,3-glucan synthase (GT48, EC 2.4.1.34) was identified in all *Trichoderma* spp., while *S. cerevisiae* and other fungi contain up to three such enzymes (13, 14). To investigate the contribution of the identified enzyme to β-1,3-glucan synthesis and, indirectly, cell wall strength, we constructed a knockout strain (Δ*fks1*) in *T. atroviride*. The deletion of *fks1* was lethal, resulting in aberrant growth and mitotic instability (Fig. S1), suggesting that *fks1* is the main enzyme involved in glucan synthesis in *T. atroviride*.

### Comparative cell wall analysis of *T. atroviride* and *T. reesei* as model organisms for mycoparasitic and saprotrophic growth

The alcohol-insoluble residue (AIR) representing the total cell walls accounted for ca. 51% and 43% of the mycelial biomass and 18% and 25% of the conidial biomass in *T. atroviride* and *T. reesei*, respectively. The carbohydrate content of these samples was further analyzed, as presented below.

#### Higher levels of glucosamine in *T. atroviride* indicate higher chitin content in the mycelial cell wall

**Fig 2:**
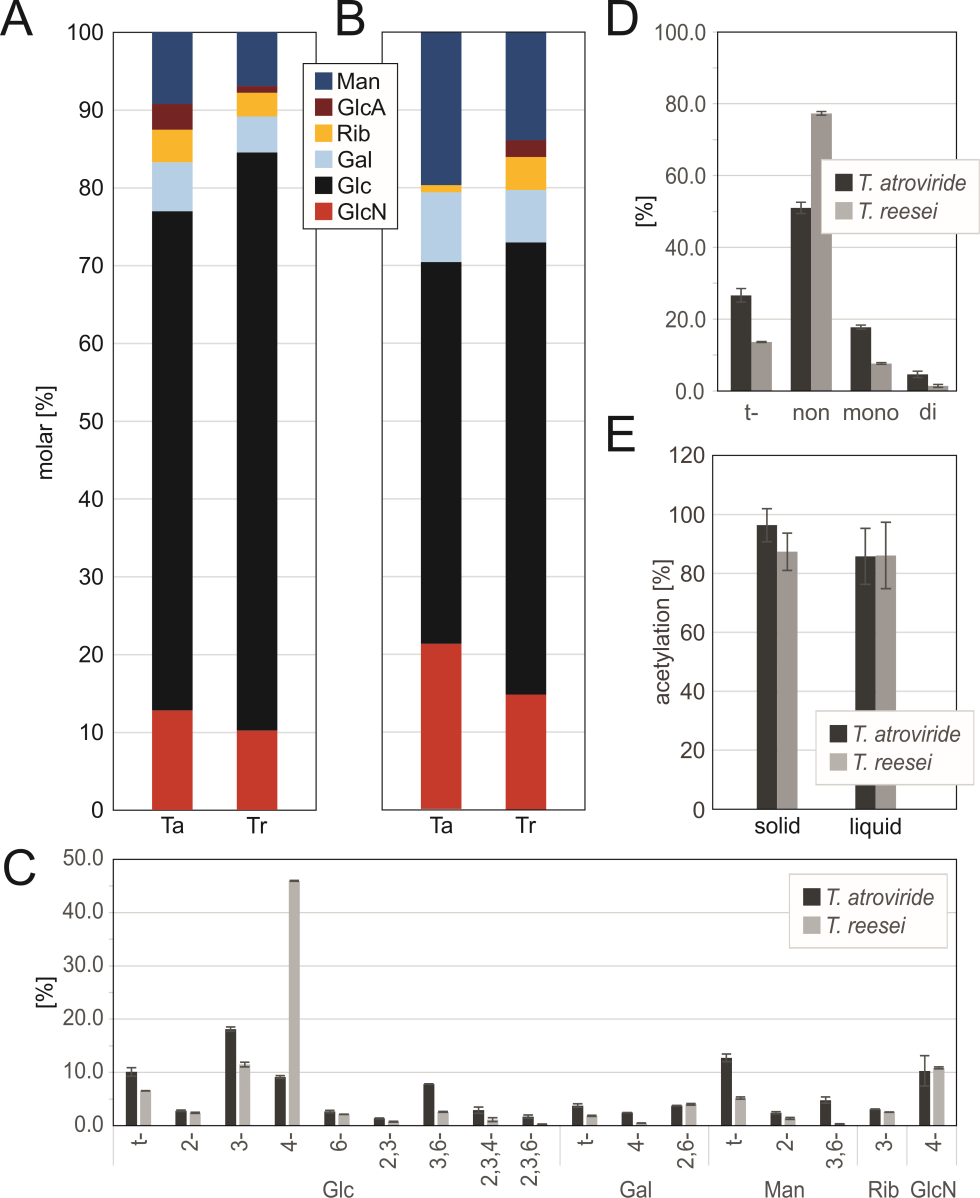
Accumulation of glucosamine in *T. atroviride* indicates elevated levels of chitin in the mycelial cell walls of mycoparasites. (**A, B**) Monosaccharide composition (mol%) of the carbohydrate fraction of the total cell walls from mycelium *T. atroviride* (Ta) and *T. reesei* (Tr) on potato dextrose agar (**A**) or in liquid shaking culture (**B**). Man, mannose; GlcA, glucuronic acid; Rib, ribose; Gal, galactose; Glc, glucose; GlcN, glucosamine. (**C, D**) Glycosidic linkage analysis (mol%) of the carbohydrate fraction of the total cell walls from *T. atroviride* and *T. reesei* grown in liquid shaking culture. The different glycosidic linkage forms of each monosaccharide were deduced from high performance liquid chromatography (HPLC) analysis. (**C**) Nomenclature used for the sugar derivatives: “t-” indicates a “terminal” monosaccharide; numbers separated by a comma indicate linkage type. (**D**) Total terminal, non-, mono-, or di-substituted linkages. (**E**) *N*-acetyl content (%) of chitin samples determined by HPLC. Values are means ± SD of two biological replicates with two technical determinations as described in Materials and Methods.

We first compared the composition of the mycelial cell walls of *T. atroviride* and *T. reesei* grown in liquid and solid cultures. Monosaccharide analysis showed that cell walls of both species have the same molecular composition, but the relative abundance of the sugar residues differs considerably between species and culture conditions (Fig. 2A and B). All samples analyzed contained glucosyl (Glc), glucosaminyl (GlcN), galactosyl (Gal), mannosyl (Man), and small amounts of glucuronosyl (GlcA) and ribosyl (Rib) residues. Glucosyl residues were dominant in all samples, representing 64.2% (± 2.3%) of the total sugars in cells grown on solid medium for *T. atroviride* and 74.3% (± 2.31%) for *T. reesei* (Fig. 2A). The Glc content in cells grown in liquid medium decreased to 49.1% (± 0.5%) and 58.11% (± 1.8%) in *T. atroviride* and *T. reesei*, respectively (Fig. 2B). Interestingly, the GlcN content in cells grown in liquid cultures was higher in *T. atroviride* (21.2% ± 1.2%) compared to *T. reesei* (14.8% ± 1%) (Fig. 2B), while no significant difference was observed in cells cultured on solid medium (Fig. 2A).

The presence of up to 19.6% (± 0.5%) Man and 9.0% (± 0.2%) Gal in mycelial cell walls suggests the presence of galactomannans and glycoproteins in *Trichoderma* spp. Indeed, the abundance of these residues also varied depending on growth conditions, with higher amounts detected in liquid cultures (Fig. 2A and B). In addition, relatively low amounts of GlcA (2%) were found in *T. reesei* and 3%–5% Rib was detected in both species and both conditions (Fig. 2A and B).

Analysis of glycoside linkage types revealed that both species are rich in non-substituted residues and contain low amounts only of derivatives corresponding to mono- and di-substituted residues (Fig. 2C and D). Interestingly, the most prominent derivative in the mycelium of *T. reesei* corresponds to 1,4-linked Glc, while this compound was low in *T. atroviride* (Fig. 2C). 1,4-Linkages are typically found in cellulose, starch, and β-(1,3;1,4)-glucans, which has recently been detected in *Aspergillus fumigatus* and *N. crassa* (29–31). In an assay for β-(1,3;1,4)-glucan, we detected ca. 1% of this type of polysaccharide (Fig. S2). Hence, another unidentified polymer containing 1,4-linked Glc residues might be present in higher amounts in the cell walls of *T. reesei*.

GlcN derivatives were essentially 1,4-linked (10%) (Fig. 2C), accompanied by minute amounts of t-GlcN (<0.1%), indicating that chitin is present in both species, as expected. Terminal, 1,4- and 1,2/1,6-linked Gal were found in the mycelium. Negligible amounts of 1,3-linked Rib were detected in both species.

Due to the significantly high levels of chitin detected in the mycelium of both *Trichoderma* spp. and the increased number of CDA in *T. atroviride* and the mycoparasitic *Trichoderma* species (Fig. 1A), we determined the degree of acetylation of chitin in the cell walls of both species. Chitin is typically crystalline and highly recalcitrant, but enzymatic deacetylation increases its solubility and flexibility (32). Chitin in the mycelium grown in liquid medium of both *Trichoderma* spp. was acetylated to ca. 85% (Fig. 2E). On solid medium, the acetylation degree of chitin was similar in *T. reesei* (86.7% ± 6.4%) but increased to ca. 97% in *T. atroviride*. Thus, up to 15% of the chitin acetyl residues are hydrolyzed by CDA in both species in liquid shaking culture and, for *T. reesei*, on solid medium, most probably rendering the polymer more flexible during active growth (32).

#### Analysis of the *Trichoderma* conidial cell walls revealed complex glycosidic linkage patterns

**Fig 3:**
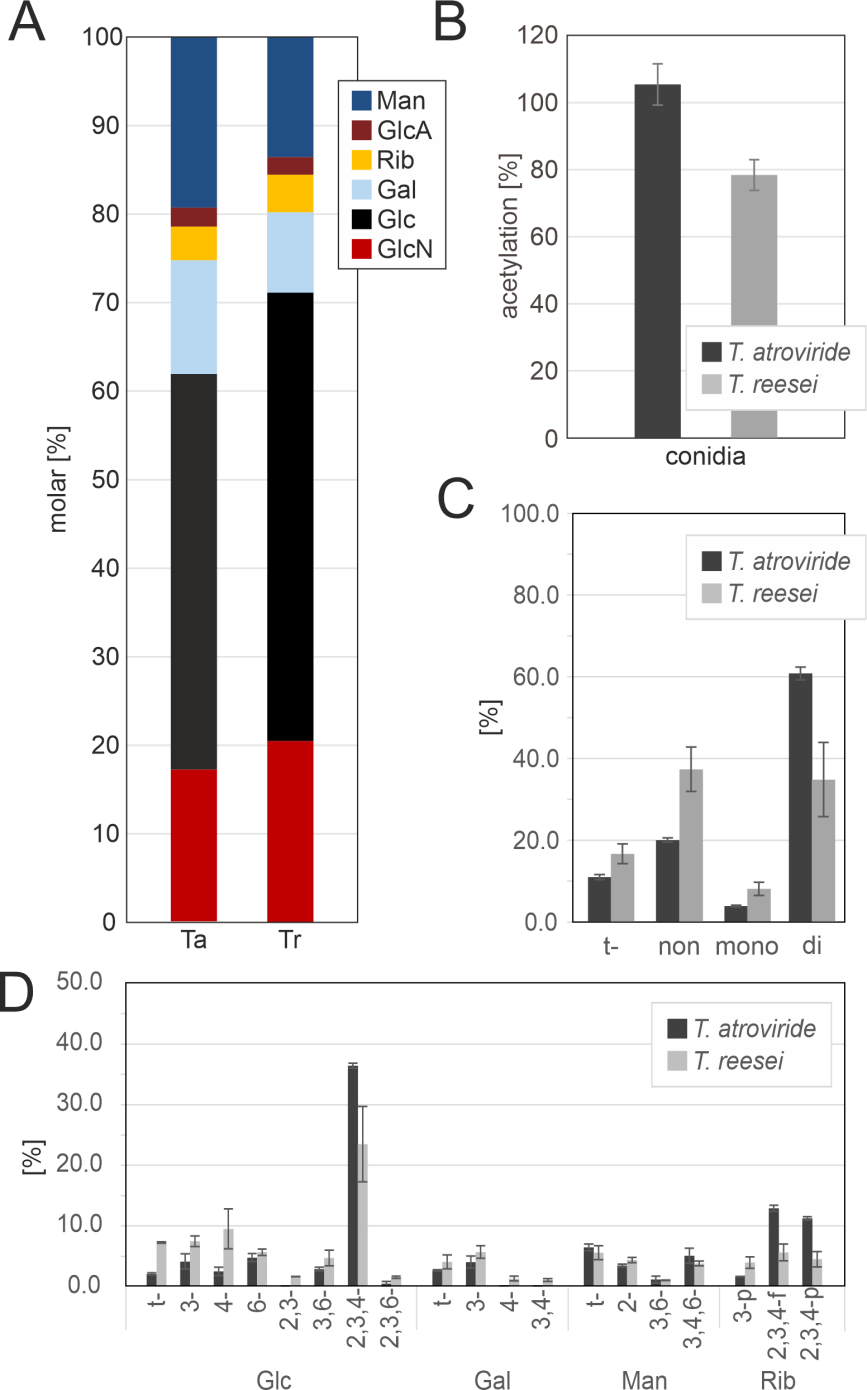
Analysis of the conidial cell walls of *Trichoderma* spp. revealed a complex polysaccharide linkage pattern. (**A**) Monosaccharide composition (mol%) of the carbohydrate fraction of the total cell walls from conidia of *T. atroviride* (Ta) and *T. reesei* (Tr). Man, mannose; GlcA, glucuronic acid; Rib, ribose; Gal, galactose; Glc, glucose; GlcN, glucosamine. (**B**) *N*-acetyl content (%) of chitin samples determined by high performance liquid chromatography (HPLC). (**C**) Glycosidic linkage analysis (mol%) of the carbohydrate fraction of the total cell walls from *T. atroviride* and *T. reesei* conidia deduced from HPLC analysis. Total terminal, non-, mono-, or di-substituted linkages are shown. (**D**) The different glycosidic linkages and pyranose or furanose forms of each monosaccharide. Nomenclature used for the sugar derivatives: “-p” and “-f” at the end indicate that the residue occurs in the pyranose or furanose form, respectively; “-” indicates a “terminal” monosaccharide; numbers separated by a comma indicate linkage type. Values are means ± SD of two biological with two technical determinations as described in Materials and Methods.

To gain insight into the compositional differences between the conidial cell walls from both species and the mycelium, carbohydrate analyses were repeated on isolated mature conidia. The AIR recovered from the conidia represented 20%–25% of the total biomass, which is significantly lower compared to the mycelial cells (40%–50%). In the conidial walls, the content of the main monosaccharides differed from the hyphal cells: in both species, the Glc levels decreased by 6%–20% whereas the amounts of Gal and Man increased by 5%–10%; however, the GlcA and Rib levels did not change significantly (Fig. 3A). Interestingly, GlcN levels were comparable, with 20.5% (± 1.6%) detected in *T. reesei* and 17.4% (± 0.9%) in *T. atroviride* (Fig. 3A). Analysis of the degree of *N*-acetylation in the conidial cell walls, however, revealed that chitin in *T. atroviride* is nearly fully acetylated, whereas its counterpart from *T. reesei* was 21.6% (± 4.6%) deacetylated, showing a slight decrease in acetylation compared to the mycelial samples (Fig. 3B).

Compared to the mycelium, linkage analysis of the conidial samples revealed a clear decrease in non-substituted glucans along with a strong increase in di-substituted types of linkages, such as 2,3,4-Glc (Fig. 3C and D). In *T. atroviride*, the increase of mono- and di-substituted residues was particularly pronounced. In addition, the Gal profile was more complex in the conidia, with terminal, 1,3-, 1,4-, and 1,3/1,4-linkages detected, suggesting the presence of highly branched galactomannans in the conidia. These polymers might arise from glycoproteins in the amorphous outer layer of the wall. Linkage analysis further showed that non-substituted 1,3-linked Rib was detected in the mycelium, whereas the conidial wall contained additional peaks indicative of the presence of 2,3,4-ribofuranosyl and 2,3,4-ribopyranosyl residues (11%–12% in *T. atroviride*, 4.5%–5.5% in *T. reesei*) (Fig. 3D).

In summary, these data highlight the increased levels of chitin in *T. atroviride* and differences in the deacetylation state of chitin as one of the strongest structural differences in cell wall carbohydrate content between the two vegetative stages of the mycoparasitic and saprophytic species. The differences observed between the conidia and mycelia of the two *Trichoderma* spp. are a further characteristic distinction of their cell walls.

### Chitin synthase activity is consistent with high levels of chitin in the mycelium of the *Trichoderma* mycoparasite

**Fig 4:**
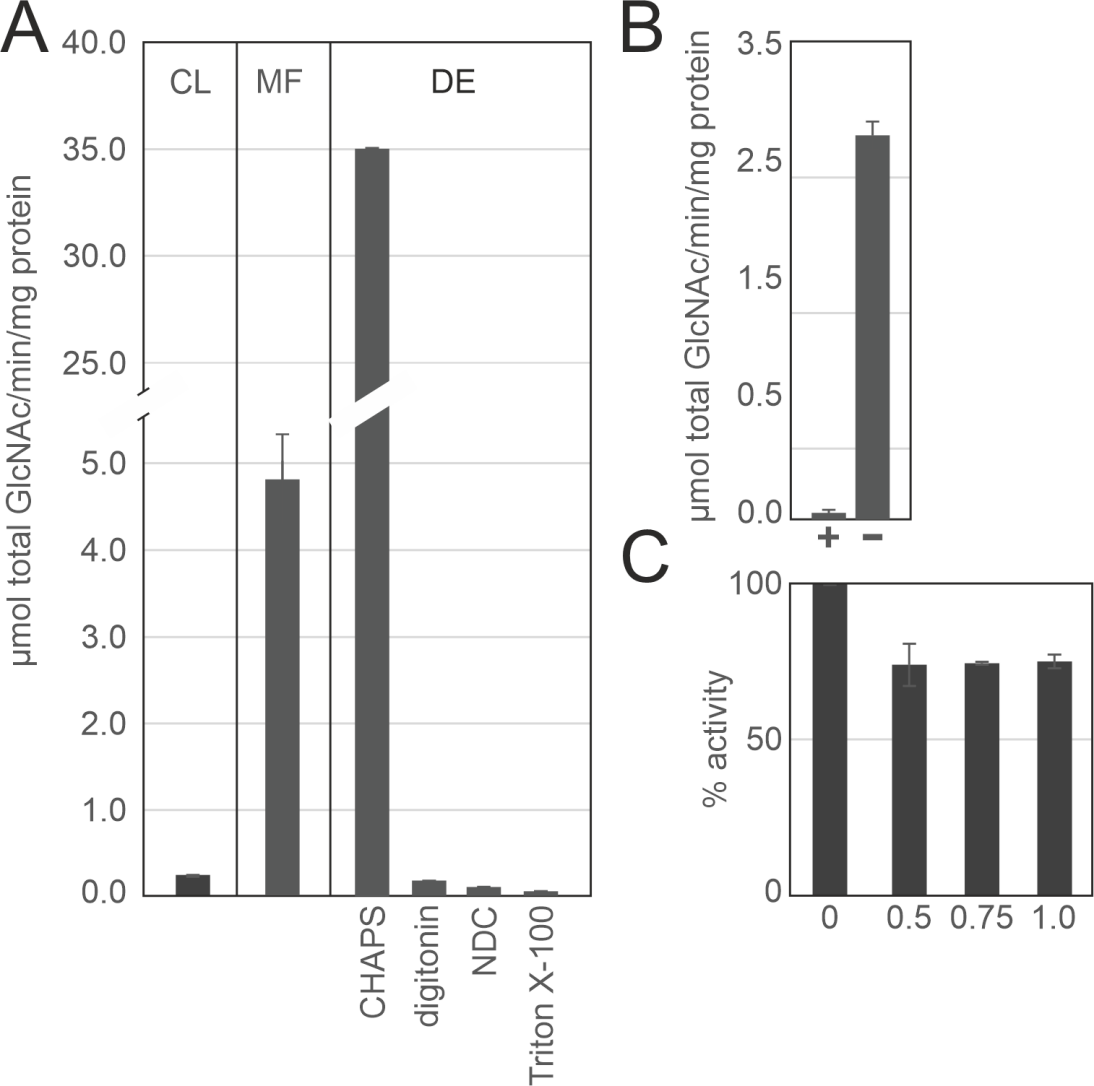
High chitin synthase activity reflects the high levels of chitin in the vegetative mycelium of the *Trichoderma* mycoparasite. (**A–C**) *In vitro* radiometric assay of chitin synthase activity measuring incorporation of ^14^C-labeled UDP-GlcNAc into insoluble chitin. (**A**) Activity of bulk chitin synthases from enzymes in the CL, the MF, or enzymes extracted from the MF [detergent extract (DE)] with the detergents 0.5% CHAPS, 1.0% digitonin, 1.5% sodium dodecylcholate (NDC), 1.0% Triton X-100. (**B**) Recovery of insoluble chitin from microsomal fractions in the presence of chitinase from *Streptomyces griseus* (≥200 units/g). (**C**) Detection of insoluble chitin from microsomal fractions in the presence of nikkomycin Z at different mM concentrations. Values are means ± SD of at least two biological with three technical determinations as described in Materials and Methods.

We previously showed that eight chitin synthases are present and active in *T. atroviride* and *T. reesei* (9). Here, we verified their presence in 13 additional species *in silico* (Table 1) and investigated whether the mycoparasitic life of *T. atroviride* is reflected in a strong chitin synthase activity during mycelial growth. *In vitro* activity in the cell lysate (CL) was low, i.e., 246 (± 11) nmol GlcNAc/min/mg protein (Fig. 4A; Fig. S3), compared to a 20-fold higher activity in the microsomal fraction (MF; 4.8 ± 0.5) µmol GlcNAc/min/mg protein) (Fig. 4A). In earlier work, enzyme activity was shown to increase during extraction from microsomal fractions by certain detergents, whereas other detergents inactivate chitin synthases during this process. Thus, three detergents were tested to determine which best preserves chitin synthase activity (33). Notably, the activity in 3-((3-cholamidopropyl)dimethylammonio)-1-propanesulfonate (CHAPS) extracts of microsomal membranes increased to 35 (± 0.12) µmol GlcNAc/min/mg protein. The other detergents tested, however, negatively impacted chitin synthase activity. The identity of the insoluble product generated in the assay was confirmed by hydrolysis in the presence of a specific chitinase, which resulted in total loss of recoverable insoluble chitin (Fig. 4B). Interestingly, nikkomycin Z, an inhibitor of chitin synthase activity, only reduced the activity by 25%, even at a concentration of 1 mM (Fig. 4C). We speculate that the active site of the enzymes may not be fully accessible to nikkomycin Z, leading to partial inhibition by the inhibitor. Altogether, these results show that the chitin synthases from *T. atroviride* are active *in vitro*.

### Mycoparasitic interaction leads to increased chitin deacetylation and chitosan deposition in *T. atroviride*

In a recent study, we observed, at the transcript level, a strong induction of specific chitin synthases and deacetylases during mycoparasitism in *T. atroviride* (9). Here, we investigated whether this is reflected by increased chitosan deposition in the cell wall, as proposed previously, to shield the hyphae from its own lytic enzymes and recognition by the host.

**Fig 5:**
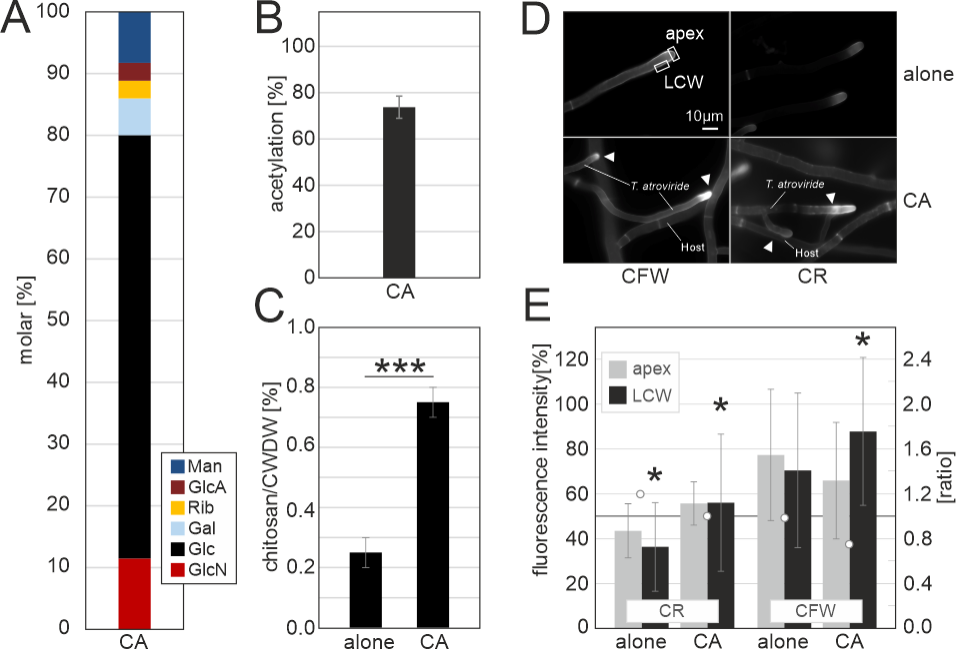
Mycoparasitic interaction leads to increased chitin deacetylation and elevated chitosan levels in *T. atroviride*. Analyses from cell walls of *T. atroviride* unchallenged (alone) or challenged (CA) with the host *S. sclerotiorum* (Fig. **S4**). (**A**) Monosaccharide composition (mol%) of the carbohydrate fraction of the total cell walls from mycelium of *T. atroviride*. Man, mannose; GlcA, glucuronic acid; Rib, ribose; Gal, galactose; Glc, glucose; GlcN, glucosamine. (**B**) *N*-acetyl content (%) in *T. atroviride* determined by high performance liquid chromatography (HPLC). Mean ± SD of two and three biological replicates and two technical replicates are shown in A and B, respectively. (**C**) Relative amounts of acetic acid extractable chitosan in % of cell wall dry weight (CWDW) of *T. atroviride*. Statistical analysis (Student’s *t*-tests) with *P* ≤ 0.01 is indicated (***). (**D**) Representative pictures of Calcofluor white (CFW) and Congo red (CR) staining of *T. atroviride* hyphal tip areas. (**E**) Semi-quantitative densitometry of fluorescence intensity of CR or CFW in the hyphal tip (apex, gray bars) and lateral cell wall (LCW, black bars) and their ratios, indicated with white circles. Statistical analysis using Student’s *t*-test with *P* ≤ 0.05 is indicated (*).

*T. atroviride* is a highly active necrotrophic mycoparasite of *Sclerotinia sclerotiorum* [(9), Fig. S5)]. No significant differences were observed between the monosaccharide composition of the cell walls of *T. atroviride* confronted with the host *S. sclerotiorum* and unchallenged mycelium (Fig. S4). Glc (68.6 ± 6.96 %) and GlcN (11.5 ± 1.65 %) levels remained comparable to the unchallenged control condition, i.e., mycelium grown on potato dextrose agar (PDA) (Fig. 5A and 2A). We asked if the induced expression of CDAs (9) would lead to increased levels of chitin deacetylation. Interestingly, directly before contact (BC) with *S. sclerotiorum,* the acetylation degree of chitin decreased to 73.7% (± 4.8) in *T. atroviride* (Fig. 5B) when compared to growth alone on solid medium with 96.6% (± 5.6) acetylation (Fig. 2E).

High performance liquid chromatography (HPLC) analysis can only inform about the overall deacetylation degree of chitin. To determine if the increase in deacetylation in *Trichoderma* is due to chitosan production, we extracted native chitosan from mycelium at the confrontation zone BC with a host as described elsewhere (34). A total of 0.25% (± 0.05) chitosan (g/g cell wall dry weight) was extracted from the hyphal walls of *T. atroviride* cultured without a host (Fig. 5C). Intriguingly, upon confrontation with *S. sclerotiorum,* the amount of chitosan increased significantly to 0.75% (± 0.05), or up to three times as much as in the unchallenged condition.

Live cell staining of chitosan in *T. atroviride* growing in the presence of *S. sclerotiorum* was performed using two different dyes to localize the sites where the increased amounts of chitosan are deposited. The fluorochrome Calcofluor white (CFW) stains β-1,4-linked glycans, and Congo red (CR) is more specific for chitin and chitosan (35). Fluorescence microscopy observation of *T. atroviride* cultured without *S. sclerotiorum* showed equal distribution of staining by CFW or CR at the subapical hyphal zone between the tip apices and the lateral walls (Fig. 5D and E). An equal and significant increase of intensity was observed in both zones with CR at the contact with the host, indicating that the deposition of chitin/chitosan increased in preparation for contact with the host. In contrast, an increase was only visible in the lateral walls but not the apices with the second, less specific cell wall dye CFW (Fig. 5D and E). We suspect that this subtle difference could not be detected in the monosaccharide analysis (Fig. 5A) since these were done on total mycelium as opposed to selected apical hyphal areas for semi-quantification with fluorescence microscopy. Altogether, the data indicate that chitosan is a structural component whose deposition in the cell wall increased close to the hyphal tips of *T. atroviride* during mycoparasitic attack.

## DISCUSSION

This study aimed to investigate the composition of the mycoparasitic cell wall by comparing two *Trichoderma* species as model organisms. The dynamic cell wall architecture of both mycoparasitic and saprophytic species as well as their vegetative development stages (mycelium and mature conidia) were examined. The availability of the genomes of the most representative species of the *Trichoderma* genus enabled precise comparative studies within the taxa. Recently, 12 of these published genomes were mined for a comprehensive evolutionary analysis (28), showing that *Trichoderma* species arose 66 million years ago (mya) and further differentiated 25–22 mya. This differentiation caused a high genomic diversity within the mycoparasitic and saprotrophic clades to adapt to their specific environmental niche. For example, gene expansions in *T. virens* and *T. atroviride* did not occur in *T. reesei,* suggesting that genes specifying mycoparasitism are present in these species (28). The ancestral mycoparasitic role of *Trichoderma* spp., together with the availability of genomic data, makes *Trichoderma* spp. ideal candidates for the first comprehensive study of cell wall composition in mycoparasites.

In a sequence homology survey of 13 *Trichoderma* spp., we identified up to 316 enzymes per species in the mycoparasitic clades, while the four species from the mainly saprotrophic clade have a significantly reduced set of enzymes, i.e., approximately 269 ± 5. As reported recently, CAZymes are comparable in terms of total gene counts versus genome size, indicating that phylogenetic distance is related to expansion in specific groups within CAZyme families (36). Interestingly, the difference between mycoparasitic vs saprophytic species is mainly due to a diversification of enzymes involved in chitin metabolism in all mycoparasites. The additional ca. 15 chitin metabolic enzymes suggest they are important in rapid cell wall adaptation during mycoparasitism. We recently demonstrated the importance of enzymes involved in chitin and chitosan synthesis and processing for *T. atroviride* mycoparasitism (9). Furthermore, the presence of elevated numbers of CHITs (GH18, EC 3.2.1) in mycoparasites (3) and their differential expression during vegetative growth and fungal attack were related to mycoparasitic capability (17, 20, 37). Our data corroborate this hypothesis, since chitinases, chitosanases, and AA7 enzymes, involved in the oxidative release of chitooligosaccharides from recalcitrant chitin, are expanded in the mycoparasitic species. These findings indicate an important role of these enzymes in mycoparasitic interaction, facilitating chitin and chitosan hydrolysis to render the host’s recalcitrant cell wall material more accessible for, e.g., nutritional purposes.

Cell wall monosaccharide and linkage analyses showed that the bulk of the exopolysaccharides is represented by 1- to 3-linked Glc and is within the range of β-(1,3)-glucans detected in most ascomycetes investigated to date (65%–90%) (11). The presence of only one GT48 enzyme for β-1,3-glucan synthesis is also a feature of other filamentous fungi (14). Additionally, a high amount of 1,4-linked glucan was detected in the mycelium of *T. reesei* (45.9%), but not *T. atroviride*. The presence of β-(1,3;1,4)-glucan in *A. fumigatus* and *N. crassa* has been described as a unique feature (29–31). Furthermore, in *Candida albicans* and some other *Candida* spp. cell wall β-glucans have been found covalently linked to glycogen (38) and serve as storage in or near the cell wall. Although our *in silico* analysis identified an enzyme with predicted β-(1,3;1,4)-glucan synthase activity in the *Trichoderma* spp., only around 1% β-(1,3;1,4)-glucan could be detected in both fungi. Increased numbers of GH13 α-1,4-glucan branching enzymes were detected in the saprophytic clade and some species of the mycoparasitic clade *in silico*. These enzymes are involved in branching and mobilizing carbohydrates from glycogen and starch-like polysaccharides (39) and could be an indication that such storage molecules are present in higher amounts in certain *Trichoderma* spp.

With 21%, *T. atroviride* may be placed among ascomycetous species with exceptionally high amounts of chitin. Although lower in *T. reesei*, chitin amounts still reach 15% and 10% in liquid and solid cultures, respectively. Furthermore, chitin synthases from *T. atroviride* appear to be active, as shown in *in vitro* activity assays. This supports previous findings of high chitin synthase expression in *Trichoderma* spp. (9). In addition, chitin seems to be of importance in harsh environmental conditions to stiffen the cell wall, as in shaken liquid cultures. In contrast to yeasts, e.g., *S. cerevisiae*, where the chitin/chitosan levels are increased in the conidia and are considered to contribute to the attachment of the tyrosine layer (1), chitin levels increased slightly in *T. reesei* and decreased slightly in *T. atroviride*. The 17–20 mole percent chitin in the *Trichoderma* conidia is already high and higher levels may not be required to promote conidial cell wall rigidity. However, chitin appears to play an important role in hyphal wall plasticity. It is tempting to infer that the increase in chitin in the mycoparasite *T. atroviride* evolved as a pathogenic trait. For example, as in dimorphic yeasts, a switch to the filamentous and infectious state is concurrent with an increase in chitin levels (15, 40).

Interestingly, linkage analysis also revealed a strong increase in branched linkage types in the conidia compared to the mycelial walls. Particularly in *T. atroviride,* an increase in mono- and di-substituted Glc, Man, and Gal residues was obvious in conidial samples. We identified a gene encoding a putative GT24 β-1,6-glycosyltransferase in *Trichoderma* spp. which might be involved in transglycosylation. Such increased cross-links might provide further physical strength to the conidial cell wall.

Unexpectedly, we also identified small amounts of ribosyl residues (0.9%–4.2 %) and linkage analysis even showed evidence for highly branched ribofuranosyl and ribopyranosyl residues. In fact, ribose has previously been identified in small amounts in cell walls of medicinal mushrooms (41–43). *Trichoderma* spp. are known for their efficient secretion system, using extracellular vesicles (EV) to export enzymes, structural proteins, lipids, and glycans. Also, several, mainly noncoding, RNA species have been detected in these vesicles and thus might be captured during transit through the cell wall (44). A first explanation could therefore be that our analysis detected ribose from the RNA present in the transiting EV. If and to what extent such vesicles transit through cell walls of mature spores remains unclear.

Protective measures by mycoparasites against detection of cell wall components by the host but also from their own defense reactions (e.g., secreted GH) still lack detailed understanding. Here, we provide the first evidence that the chitinous polysaccharides in the two analyzed *Trichoderma* spp. cell walls are ca. 5%–15% deacetylated in the vegetative mycelium and ca. 0.25% of the cell wall dry weight is chitosan. These findings suggest that a certain proportion of residues in chitin is deacetylated to render the polymer more flexible and less recalcitrant (32). Protective strategies in plant pathogenic fungi have been studied, e.g., mannans and α-glucan can serve as a disguise in these fungi (10). Conversion of surface-exposed chitin to chitosan, by activating cell wall CDA, is another efficient strategy used by plant pathogens. Chitinases cannot act on chitosan, which is a weak activator of the plant immune response (23, 45). Interestingly, in confrontation with the host *S. sclerotiorum,* the degree of acetylation of chitin decreased significantly in *T. atroviride* to only 75% with a concomitant threefold increase in chitosan (0.75%), and increased chitin/chitosan staining at the tips and lateral walls of *T. atroviride*. The high deacetylation in the mycoparasite challenged with a host is corroborated by the concurrent activation of a set of CDA and chitosanases that are specific to the mycoparasitic clade of the investigated species (9). In addition, single knockout *cda* strains were severely compromised in mycoparasitic overgrowth of the host even though they produced increased amounts of mycoparasitism specific enzymes (ECH42, NAG1, PRB1). Interestingly, the stalled growth upon contact with the host seemed to be due mainly to an increased susceptibility toward host-derived secreted secondary metabolites and enzymes (9). Thus, the elevated levels of chitosan suggest that *Trichoderma* evolved chitin deacetylation as a means to hide from host detection during mycoparasitic attack. Recently, deacetylation of released chitin oligomers from *Verticillium dahliae* during infection of cotton was shown to be part of an efficient virulence strategy by the pathogen to circumvent detection and immune responses by the plant (46). By contrast, a direct deacetylation of the chitin present in *V. dahliae* cell walls could not be verified. Thus, we hypothesize that, in contrast to this plant pathogen, *T. atroviride* and possibly other mycoparasites might have evolved direct deacetylation of the exposed cell wall chitin as an efficient strategy to circumvent host defense. Our findings corroborate our hypothesis that the expansion of chitosan processive enzymes in mycoparasitic species and their elevated expression upon contact with a host (9) are beneficial to the mycoparasitic attack. Consequently, it will be important to determine if chitosan can be sensed by plant pathogens or if, similar to plants (23, 45), chitosan acts as a blind spot.

In summary, we present a detailed *in silico* analysis of the enzymatic repertoire of 13 *Trichoderma* spp. related to cell wall metabolism. The analytical and biological analysis of the cell walls of two representatives, the mycoparasite *T. atroviride* and the predominantly saprophyte *T*. reesei, reveals a concise picture of the dynamic cell wall composition of *Trichoderma* spp. in different culture conditions and vegetative life stages. Our analysis provides insights into the mycoparasitic cell wall architecture. An expanded set of chitin active enzymes, high levels of chitin, and its dynamic adaption during environmental changes set the mycoparasitic *Trichoderma* spp. apart from the mainly saprotrophic species. The determination of the degree of acetylation and the low but significant amounts of chitosan in the cell wall support our previous hypothesis (9) that a higher amount of chitin and its partially deacetylated form chitosan are beneficial for mycoparasitic *Trichoderma* spp. during host interaction. These findings might apply to other mycoparasites and help in optimizing fungal biocontrol and production systems.

## MATERIALS AND METHODS

### Strains and cultivation conditions

*T. atroviride* IMI206040 [teleomorph: *Hypocrea atroviridis*, (https://mycocosm.jgi.doe.gov/Triat1/Triat1.home.html] and *T. reesei* QM6a were maintained on PDA (BD, Franklin Lakes, USA), and incubated at 28°C with a 12-h/12-h light cycle. The fungal host *Sclerotinia sclerotiorum* was cultivated as described previously (9, 47, 48).

### Generation of *fks1* knockout mutants

*T. atroviride fks*1 knockout mutants were generated as described elsewhere (9). Briefly, primers HY and YG were used to amplify the *Escherichia coli* hph-phosphotransferase marker cassette (9). Primers (F1, F2, F5, and F6, Table S8) were used to amplify 1.5 kb fragments of upstream and downstream regions flanking the 5,772 bp *fks1* open reading frame (jgi_triat2_140247). The flanking regions were combined with the hph-phosphotransferase marker cassette in a double joint PCR reaction to create two constructs with a ca. 1,000 bp overlap for use in protoplast transformation with the split marker technique (49, 50). The transformants were recovered from PDA plates containing 200 µg/mL hygromycin B and subjected to several rounds of single spore isolation. The integration of the construct was verified using the primer pairs F3 and F4 (Table S8; Fig. S1).

### Chemicals and kits

Chemicals were obtained from Roth (Carl Roth GmbH + Co. KG, Karlsruhe, Germany) and Sigma-Aldrich (St. Louis, MO).

### Culture conditions

Liquid minimal culture medium (Synthetic minimal (SM)) was used for shake flask cultivation (250 rpm) at 28°C in the dark, as described previously (9). The mycelium was harvested after 72 h by filtering through miracloth (Millipore/Sigma, VWR), followed by washing with distilled water.

Mature conidia of *T. atroviride* and *T. reesei* were harvested from 7-day-old cultures on PDA after incubation in darkness until the plates were covered with mycelium (ca. 72 h) followed by 12-h/12-h light-darkness cycles to induce synchronized sporulation.

Dual confrontation assays of *T. atroviride* against *S. sclerotiorum* were performed as described in reference (47). Briefly, the surfaces of PDA plates were covered with autoclaved cellophane to facilitate the harvesting of mycelium. Agar pieces bearing the mycelium of *T. atroviride* and the host were placed on a PDA-cellophane plate 50 mm apart from each other. The plates were subsequently incubated at 28°C until the hyphae of both fungi were 1–2 mm apart (BC). From each plate, 5 mm of the mycelial periphery of *T. atroviride* was harvested. Peripheral mycelium of *T. atroviride* grown in the absence of the host served as control. All harvested mycelia were freeze-dried and stored at room temperature until further processing.

For cell wall analysis, harvested mycelia and spores were treated with ethanol (99.9%, Roth) and homogenized with an Ultraturrax (IKA-Werke GmbH & Co. KG, Staufen, Germany). The resulting cell walls were washed with excess cold water and freeze-dried for further analysis.

### Determination of cell wall composition

Fifty milligrams of intact mycelium was used to obtain AIR comprising the purified cell walls. AIR were subsequently treated in the presence of the *Bacillus* thermostable α-amylase/amyloglucosidase to remove starch-like glycans. Three independent measurements (monosaccharide analysis, chitin degree of deacetylation, and glycosidic linkage) were conducted independently to characterize the composition of the extracted fungal cell walls.

#### Monosaccharide analysis

Determination of monosaccharide composition of the fungal cell walls were conducted from 10 mg of freeze-dried AIR samples by hydrolysis with 13.4 M H_2_SO_4_ for 3 h at room temperature (RT) and further treatment with 2 M H_2_SO_4_ for 3 h at 100°C. The resulting monosaccharides were diluted with distilled water and derivatized with 1-phenyl-3-methyl-5-pyrazolone at 70°C for 1 h and analyzed by reversed-phase HPLC using a Phenomenex Kinetex 2.6 µm C18 100 Å column (100 mm × 3 mm) (Phenomenex, Torrance, USA) and an Agilent 1260 HPLC system (Agilent Technologies, Santa Clara, USA) (51). Ten microliters of sample was injected at a flow rate of 0.8 mL/min and 30°C. Elution was performed using (A) 10% acetonitrile, 40 mM ammonium acetate (pH ~6.8), and (B) 70% acetonitrile with gradients of 8%−17% B (0–9.3 min), 17%−90% B (9.3–10.0min), 90% B (10.0–11.0 min), 90%−8% B (11.0–11.5 min), and 8% B (11.5%−14.5%). Absorbance was measured at 250 nm. Solutions containing standards (Xyl, Ara, Man, Glc, Gal, Rha, Rib, GlcA, and GalA) in three different concentrations were used to obtain standard curves based on peak areas. Retention times and peak areas of samples were compared to those of monosaccharide standards for qualitative and quantitative analysis.

#### Determination of degree of deacetylation of chitin

The deacetylation degree of chitin was determined based on the amount of acetic acid and GlcN produced after hydrolysis of AIR with H_2_SO_4_ (see Monosaccharide analysis section). After hydrolysis, samples were diluted with water and acetic acid content was determined using a Rezex RFQ-Fast Acid H+ (8%) Ion Exclusion HPLC column (100 × 7.8 mm) (Phenomenex, Torrance, USA) and an Agilent 1260 HPLC system (Agilent Technologies, Santa Clara, USA). Fifty microliters of sample was injected and the flow rate of the mobile phase (5 mM H_2_SO_4_) was 0.5 mL/min (isocratic elution). Standard solutions of acetic acid at concentrations ranging from 100 µM to 1,000 µM were used to prepare a standard curve for quantitative analysis. The GlcN content (µM) was determined as indicated above (see Monosaccharide analysis section). The degree of deacetylation of chitin/chitosan (DD) was calculated as DD = 1 − (acetic acids / GlcN) * 100%.

#### Glycosidic linkage analysis

Glycosidic linkage analysis was performed as described by Pettolino et al. (52, 52), with minor modifications. Briefly, 1 mg of freeze-dried AIR was dissolved in NaOH/anhydrousdimethylsulfoxide (DMSO) and methylated by adding 100 µL of methyl iodide under N_2_. The reactions were sonicated for 10 min at 25°C and the methylation step was repeated four times. After addition of 1 mL of dichloromethane (DCM), the methylated polysaccharides were extracted by phase partitioning against deionized water. The resultant DCM phase was evaporated under a N_2_ stream, followed by hydrolysis at 100°C for 3 h in 1 mL of 2 M trifluoroacetic acid under N_2_. The hydrolysates were reduced overnight at RT in the presence of NaBD4 and subsequently acetylated with acetic anhydride at 100°C for 12 h to obtain partially methylated alditol acetates (PMAAs). The PMAAs were recovered by evaporating the acetic anhydride solvent under a N_2_ stream, and redissolving the samples in DCM. The PMAAs were further purified by partitioning against deionized water three times, and dried under N_2_. The samples were then dissolved in 200 µL of DCM and analyzed on an Agilent 7890B/5977B GC-MS instrument (Agilent Technologies, USA) fitted with a VF-23 ms capillary column (30 m × 0.25 mm, 0.25 µm, Agilent Technologies, USA). Helium was used as the carrier gas and the oven temperature was programmed as follows: from 165°C to 175°C at 1 °C/min; from 175°C to 195°C at 0.5 °C/min; from 195°C to 210°C at 2 °C/min, and from 210°C to 250°C at 10 °C/min, followed by a plateau at 250°C for 6.5 min. The fragmentation patterns of the different PMAAs were interpreted by referring to the CCRC Spectral Database for PMAAs (https://glygen.ccrc.uga.edu/ccrc/specdb/ms/pmaa/pframe.html).

### Preparation of microsomal fractions and detergent-extracted (DE) membrane proteins

Microsomal fractions were prepared as described previously (33) from fresh mycelium grown in liquid culture for 24 h in SM medium. The mycelium was homogenized in Tris-HCl 10 mM, pH 7.0 with 1× Complete EDTA-free protease inhibitor cocktail (Roche, Basel, Switzerland) and 1 mM dithiothreitol (DTT). Cells were lysed by passage through a French press with a maximum pressure of 25 MPa. Cell debris were removed by centrifugation (8,000 *g*) and MF were pelleted by centrifugation (1 h, 100,000 *g*). For enzymatic activity measurements, the final protein concentration of the MF was adjusted to 5 mg/mL with extraction buffer (Tris-HCl 10 mM, pH 7.0 with 10% glycerol). For extraction of membrane proteins, one of the following detergents was added to the MF (all from Sigma-Aldrich), i.e., CHAPS, digitonin, sodium deoxycholate, or Triton X-100 at final concentrations of 0.5 to 1.5% (wt/vol). The proteins were solubilized for 30 min with gentle stirring at 4°C. The samples were centrifuged for 1 h at 100,000 *g* and the supernatant containing the DE was used for chitin synthase activity measurements.

### Chitin synthase assay

The *in vitro* chitin synthase assay was performed as described previously (33) in an optimized buffer system for *T. atroviride* (HEPES pH 6.5). Fifty micrograms of proteins from the crude CL or the MF or 10µg of the detergent extracted membrane proteins (DE) was used as a source of enzyme for the assay (total volume of 200µL). Protein concentration was determined using the Bradford assay. The final concentrations of the components in the reaction mixtures were 0.5mM UDP-N-acetyl-D-glucosamine, 0.066nM [^14^C]-UDP-N-acetyl-D-glucosamine (glucosamine; 300 mCi/mmol; Perkin Elmer), 20mM GlcNAc, 10mM MgCl_2_, 10mM HEPES·NaOH pH 6.5, and 1mM DTT. After 1 h incubation at RT, the reactions were stopped by the addition of 400µL 95% ethanol, and the insoluble product was precipitated at −20°C. The product was recovered on glass-fiber filters (Whatman GF/C) and washed with 66% ethanol and water under vacuum. The radioactivity incorporated in the insoluble chitin was measured in a scintillation counter (Packard 1500 Tri-Carb) using 4mL Ultima Gold F scintillation cocktail (53, 54). Enzymatic activity was expressed micromole of GlcNAc incorporated into chitin per milligram of protein. Standard deviations were calculated from two biological and two technical replicates.

### Enzymatic characterization of the *in vitro* product generated in the chitin synthase assay

The product synthesized *in vitro* (section Chitin synthase assay) was characterized as described previously (33). Following centrifugation (10,000 *g*, 10 min), the product was washed with 1 mL 75% ethanol and resuspended in 200 µL 50 mM potassium phosphate buffer (pH 6.0) containing 200 µg/mL chitinase from *Streptomyces griseus* (≥200 units/g, Sigma-Aldrich) and incubated for 2 h at RT. The hydrolysis was terminated by the addition of 400 µL 95% ethanol. Resuspended samples devoid of chitinase served as controls. The non-hydrolyzed polysaccharides present in all samples were then precipitated at −20°C, recovered on glass-fiber filters, and quantified by scintillation counting as indicated above.

### Semi-quantification of chitin and chitosan

Fungal strains were grown as co-cultures on PDA until direct contact between the mycelia of *T. atroviride* and *S. sclerotiorum* was observed. To quantify changes in the peripheral confrontation zone from the control condition (*T. atroviride* alone on a plate, Fig. S4.) or at contact with the host, single hyphae were imaged using fluorescence microscopy. A dissected agar plug from the confrontation zone was submerged in a droplet of 30 µL CFW stain (20 µM, Sigma #F3543) with the inverted agar method (55) and imaged with an inverted Zeiss Axio Observer Z1 (Zeiss, Oberkochen, Germany) with differential interference contrast optics and 405 nm excitation/430–470 nm emission. Visualization and semi-quantification of *Trichoderma* cell wall chitin were performed using the following cell wall stains: CFW M2R at a final concentration of 2 µM to label β-1,4-glucans including chitin, and CR (Sigma #C6767) at a final concentration of 50 µM to specifically label chitin (35). Fluorescence signals were assessed using CFW and CR. All samples were incubated for 5 min before imaging and measured by densitometry using the MacBiophotonics ImageJ work package (https://www.macbiophotonics.ca/software.htm) as described in reference (35). Defined rectangular areas were measured in the Spitzenkörper and the lateral cell walls of the peripheral contact zone. On average, 40–100 analyses were performed per hyphae.

### Chemical extraction of chitosan from confrontation assays

Extraction of chitosan was performed twice from the mycelium at the periphery of *T. atroviride* alone and 1 mm before contact with *S. sclerotiorum*. For each extraction, a total of 60 plates were prepared for the harvest of the confrontation zones and 30 plates for mycelium from *T. atroviride* alone. Harvested mycelia from each condition were pooled for further processing. The lyophilized *T. atroviride* mycelium was pulverized using a mortar and pestle and the dry biomass weight was documented. Cell lysis was achieved by resuspending the ground mycelium in 0.1% SDS with an Ultraturrax. Soluble cell debris were removed by centrifugation (10,000 rpm, 20 min). The resulting pellet was washed by resuspension in deionized water and centrifugation until a clear supernatant was obtained. Furthermore, the protocol introduced by reference (34) for the chemical extraction of chitosan from shellfish was applied: briefly, the washed pellet was deproteinated by incubation in 2% (m/wt) NaOH overnight at RT. The obtained chitin/glucan matrix was recovered by centrifugation and washing as described above and subsequently subdued to a demineralization step with 2% (m/vol) HCl for 30 min at room temperature under stirring. To avoid losses in chitosan yield due to acidic pH, the suspension was set back to an alkaline pH (10, 11) with 50% NaOH immediately after demineralization. The overall chitin/glucan matrix was recovered by centrifugation and freeze-dried to document the resulting dry weight. Intrinsic chitosan present in the obtained chitin/glucan matrix was recovered in 2% (vol/vol) acetic acid overnight at 70°C under vigorous stirring. The acidic chitosan solution was then separated from the insoluble chitin/glucan fraction by centrifugation (10,000 rpm, 20 min). Precipitation of chitosan from the acidic solution occurred at pH 10–11 by adding NaOH while stirring, and the chitosan pellet recovered via centrifugation. All extracted chitosans were washed with 50% (vol/vol) ethanol (34) until a neutral pH of the wash was reached. The chitosan yields were determined after freeze-drying the final product.

### Bioinformatics and statistical analyses

For a comprehensive insight into the repertoire of cell wall enzymes in *Trichoderma* spp., we used the genomic data from the automated and manually curated annotation at Joint Genome Institute (JGI) (https://mycocosm.jgi.doe.gov) from 14 different *Trichoderma* spp. (Table S1) and categorized them into carbohydrate-active enzymes [(56) http://www.cazy.org/] or into Pfam categories (57) where applicable (“other”) as suggested by Muszewska et al. (12). Phylogenetic analyses were performed in Mega11 (58), using Neighbor joining as distance algorithmic method. The stability of clades was evaluated by 500 bootstrap rearrangements. Structure/function prediction was performed using InterProScan (59). At least two biological and two technical replicates were used for statistical analysis.

## SUPPLEMENTAL MATERIAL

Fig. S1 spectrum.03495-23-s0001.pdf Gel electrophoreses of PCR products generated.

Fig. S2 spectrum.03495-23-s0002.pdf Determination of β-1,3-1,4-glucan in percent (%) of the total cell wall dry weight.

Fig. S3 spectrum.03495-23-s0003.pdf SDS-PAGE showing the CL and the MF.

Fig. S4 spectrum.03495-23-s0004.pdf Harvested mycelium areas for the determination of chitosan in cell walls.

Fig. S5 spectrum.03495-23-s0005.pdf Trichoderma mycoparasitism of *Sclerotinia sclerotiorum*.

Supplemental file spectrum.03495-23-s0006.docx Description and analysis of the enzymes and fungal species examined.

Supplemental material spectrum.03495-23-s0007.docx Supplemental figure legends.

Supplemental tables spectrum.03495-23-s0008.xlsx Tables S1 to S8.

ASM does not own the copyrights to Supplemental Material that may be linked to, or accessed through, an article. The authors have granted ASM a non-exclusive, world-wide license to publish the Supplemental Material files. Please contact the corresponding author directly for reuse.
