# Supplementary figures and images for: A comparative cell wall analysis of Trichoderma spp. confirms a conserved polysaccharide scaffold and suggests an important role for chitosan in mycoparasitism
Source: Microbiol Spectr. 2024 Jun 25;12(8):e03495-23. doi: 10.1128/spectrum.03495-23 (PMC11302013; doi:10.1128/spectrum.03495-23)

1 2 3 4

6 kb

3 kb

1 kb

\*

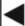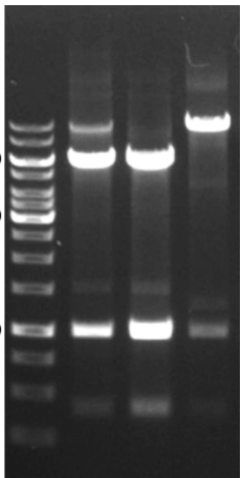

Supplement: Fig. S1 — Gel electrophoreses of PCR products generated. [file spectrum.03495-23-s0001.pdf]

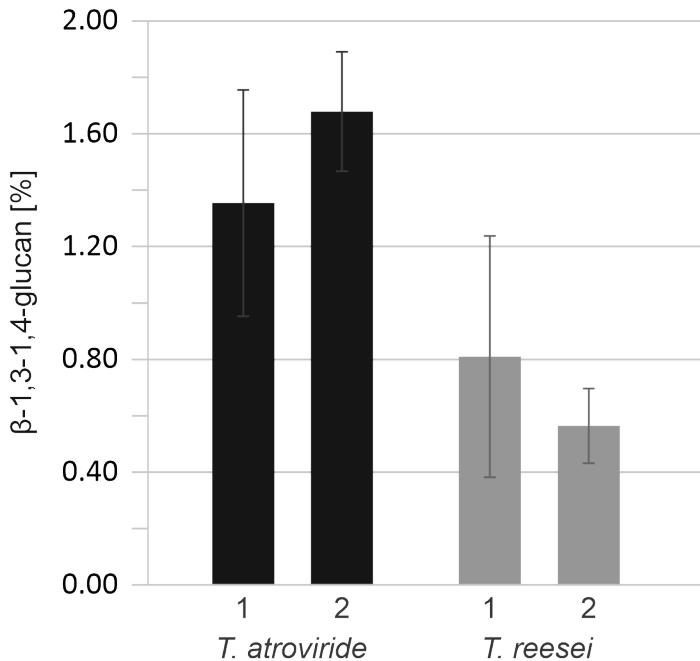

Supplement: Fig. S2 — Determination of β-1,3-1,4-glucan in percent (%) of the total cell wall dry weight. [file spectrum.03495-23-s0002.pdf]

CL MF DE RD

250 -

150 -

100 -

**75 -**

50 -

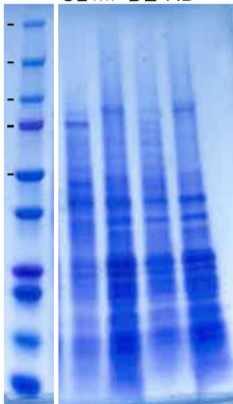

Supplement: Fig. S3 — SDS-PAGE showing the CL and the MF. [file spectrum.03495-23-s0003.pdf]

A

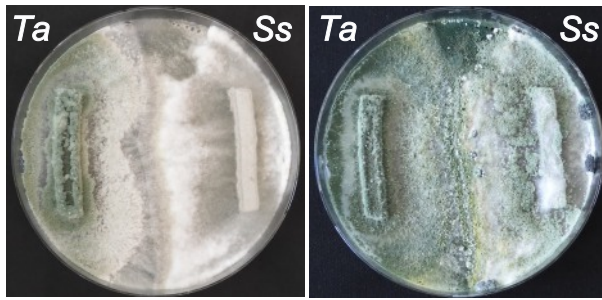

B

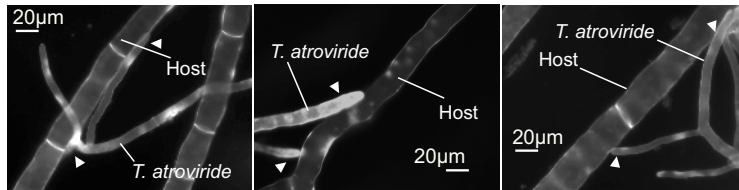

Supplement: Fig. S5 — Trichoderma mycoparasitism of Sclerotinia sclerotiorum. [file spectrum.03495-23-s0005.pdf]
