# Supplementary material for: A comparative cell wall analysis of Trichoderma spp. confirms a conserved polysaccharide scaffold and suggests an important role for chitosan in mycoparasitism
Source: Microbiol Spectr. 2024 Jun 25;12(8):e03495-23. doi: 10.1128/spectrum.03495-23 (PMC11302013; doi:10.1128/spectrum.03495-23)
Supplement: Fig. S4 — Harvested mycelium areas for the determination of chitosan in cell walls. [file spectrum.03495-23-s0004.pdf]

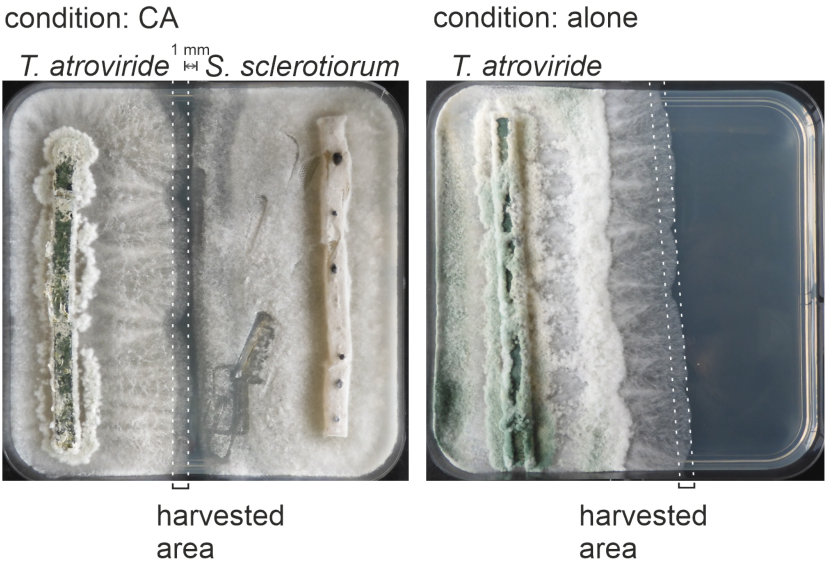
condition: CA
condition: alone
1 mm
T. atroviride
S. sclerotiorum
T. atroviride
harvested area
harvested area
