# Supplementary material for: A comparative cell wall analysis of Trichoderma spp. confirms a conserved polysaccharide scaffold and suggests an important role for chitosan in mycoparasitism
Source: Microbiol Spectr. 2024 Jun 25;12(8):e03495-23. doi: 10.1128/spectrum.03495-23 (PMC11302013; doi:10.1128/spectrum.03495-23)
Supplement: Supplemental file — Description and analysis of the enzymes and fungal species examined. [file spectrum.03495-23-s0006.docx]

Cell wall proteins in Trichoderma spp.

Contents

[Enzymes present in the *Trichoderma* spp. 1](#_Toc117868811)

[CAZy category Glycosyl Transferase (GT) 2](#_Toc117868812)

[Glycoside Hydrolase (GH) 2](#_Toc117868813)

[Carbohydrate Esterases 3](#_Toc117868814)

[Auxiliary Activity 3](#_Toc117868815)

[Pfam category 4](#_Toc117868816)

[PF00080 and PF00199 4](#_Toc117868817)

[PF00399 and PF12454 4](#_Toc117868818)

[Phylogenetic analysis of the chitin deacetylases and chitosanases in the *Trichoderma* spp. 5](#_Toc117868819)

[References: 5](#_Toc117868820)

# Enzymes related to cell wall metabolism in *Trichoderma* spp.

To get a concise picture of the gene inventory of the most abundant *Trichoderma* species, fourteen analyzed Trichoderma genomes were mined from the jgi-data base (<https://mycocosm.jgi.doe.gov>) for the presence of the proposed protein categories that are connected to cell wall metabolism. A recent study revealed the specific genomic features of 12 of the most abundant *Trichoderma* spp. for only those enzymes, which are involved in the degradation of host fungi and dead plant material [1]. Moreover, Muszewska et al., [2] resolved the gene inventory involved in cell wall metabolism in pathogenic filamentous fungi. A comparison of the data from the two publications shows that there is a considerable overlap between enzyme families for external cell wall degradation and cell wall maintenance of the own fungal cell wall of a selected species. Thus, in our analysis of the *Trichoderma* cell wall protein inventory, we used the proposed categorization of Muszewska et al., to identify CW related CAZy-families ([3], <http://www.cazy.org/>) and the mentioned Pfam protein families [4] from [2]. We screened *T. atroviride* [5], *T. gamsii* [6], *T. asperellum* [7], *T. hamatum* [8], and *T. asperelloides* [1] from the proposed mycoparasitic clade ‘Trichoderma’ (ST) [1] and *T. harzianum* [7], *T. guizouense* [7], *T. atrobrunneum* [9], *T. virens* [5] and *T. brevicompactum* [10] from the mycoparasitic clade ‘Harzianum and Virens’ (HV). We compared the two mycoparasitic clades with representatives of the clade ‘Longibrachiatum’ (SL) comprised of species with a primarily saprotrophic life style: *T. reesei* [11], *T. longibrachiatum* [7],*T. citrinoviride* [7], *T. parareesei* [12].

In addition to the strains investigated by Kubicek et al., [1] we included *T. asperelloides and T. brevicompactum* because these genomes were recently published and provide a deeper insight in the respective mycoparasitic clades. On the other hand, we were not able to include the genome of *T. hamatum.* Although an extensive analysis of its genome, (available with the GenBank accession number ANCB00000000.2 at the NCBI database), was conducted to supplement missing parts by Kubicek et al., [1], the data has not been updated yet on the jgi-database. Data on glycosyltransferases in particular are still lacking.

In our analysis we were thus able to assign 3,765 enzymes to cell wall metabolism in the thirteen selected *Trichoderma* species. From the CAZy-family with their primary function as glycosyl transferases, glycoside hydrolases, carbohydrate esterases and enzymes with auxiliary activities 78 subfamilies were identified, based on their predicted function in cell wall metabolism. In the following subsections the involved related CAZy- and Pfam protein families are discussed in detail.

### CAZy category Glycosyl Transferase (GT)

For the biosynthesis of cell wall polysaccharides, the action of glycosyltransferases (GTs) (EC 2.4.x.y) is vital. These enzymes use activated donor monosaccharides, which they transfer onto specific acceptor molecules, forming glycosidic bonds and generating homo- and heteropolysaccharide chains. We recently provided detailed analysis of this nucleotide-sugar pyrophosphorylase family and the nucleotide-sugar interconverting enzymes, which can convert an activated monosaccharide into another activated form [13], and therefore these results will not be repeated here. Note, that polyspecificity (enzymes with different donor and/or acceptor found in the same family) is common among the GT-families, so that a second detailed manual analysis of some of these groups was necessary, to increase the reliability of the predictions.

We identified 1,027 genes falling into 29 GT-families in the *Trichoderma* genomes. Interestingly, we could not identify α-1,3-fucosyltransferases (GT10, EC 2.4.1.152) nor beta-1,4-N-acetyl-galactosaminyl transferases (GT12, EC 2.4.1.92). This observation is backed by our recent study, were we only identified genes related to UDP-Glc, UDP-GlcNAc, UDP-Gal, GDP-Man, and UDP-GlcA in Hypocreaceae but genes for synthesis of GDP-fucose, UDP-arabinose, or UDP-rhamnose are missing in the *Trichoderma* spp. [13].

Interestingly, we could also not identify GT-families for α-1,3-glucan synthases (GT5, EC 2.4.1.183) which is a hint, that the fungi might not be able to synthesize α-linked-glucans.

Remarkably, our results show that three species from the mycoparasitic SV group have a gene duplication within the GT2-subfamily for chitin synthases (CHS). A gene duplication in this family in Sordariomycetes is rare but has been shown previously for *Blastomyces dermatidis* and *Trichophyton rubrum* [2]. Similar to this report *T. harzianum* and *T. guizhouense* have a duplicated chitin synthase gene in the ClassI/ClassII CHS. Interestingly though, in *T virens* the additional chitin synthase is most closely related to the recently described *chs8* gene of *T. atroviride* [14] and might stem from a duplication event of the whole gene locus, since also the downstream chitin deacetylase (CE4, see section below, (*cda1*) is duplicated in *T. virens*. The CHS-subfamily is also related to another interesting subfamily within GT2, representing putative hyaluronan like synthases (EC 2.4.1.212) and the investigated *Trichoderma* spp. harbor additional 1-3 hyaluronan like synthases in their genomes.

Remarkably, all 13 genomes harbor at least one GT2-gene encoding a putative β-1,3-1,4-glucan synthase (exception: *T. asperelloides*, which harbors three). Although we detected considerable amounts of 1,4 linked glucose a further biochemical characterization could only identify the presence of ca. 1% of β-1,3-1,4-glucan in *T. atroviride* or *T. reesei* experimentally (see main manuscript).

### Glycoside Hydrolase (GH)

We identified 2,355 genes falling into 36 GH-families in the *Trichoderma* genomes. Glycoside hydrolases (EC 3.2.1.-) are a widespread group of enzymes, which hydrolyze the glycosidic bond between two or more carbohydrates or between a carbohydrate and a non-carbohydrate moiety. By contrast to the glycosyl transferases the classification does not reflect their structural features. Moreover, most of the enzymes from these groups can be associated to more than one cellular process, since many of them are most probably involved in a) catabolic pathways [1], to extract carbon sources from complex nutritional resources, but also in b) anabolic processes [2, 15] being involved in remodeling events, i.e., by opening a polysaccharide chain at a distinct position to attach another polysaccharide, protein or lipid to the former chain.

In *Trichoderma* spp. especially the number of chitinases from the GH18 family is highly expanded and can reach a maximum of 34 enzymes in the mycoparasitic *clades,* as has been shown previously [1, 5, 16]. But also, other glycoside hydrolases are expanded in *Trichoderma* spp. (GH3 β-glucosidases, GH55 β-1,3-glucanase, GH16 endo-1,3-β-glucanase and GH71 α-1,3-glucanase) and we refer the reader for a detailed analysis for most of the GH family genes involved in carbon catabolism to [1].

We want to point out, that the GH75 chitosanases show increased numbers in the mycoparasitic species with on average 5 enzymes in the two mycoparasitic clades and 3 in the saprotrophic clade. Although genes involved in polysaccharide decomposition by *Trichoderma* have recently been evaluated [1], we noticed in our detailed manual analysis that some of the chitosanases were not included in this analysis, and we added these now to complete the analysis ([14] and this study).

### Carbohydrate Esterases

Only 2 groups of the carbohydrate esterases have been assigned to cell wall metabolism: CE4 and CE9, where we identified 74 genes in the thirteen *Trichoderma* genomes. Generally, they catalyze the de-N-acylation of substituted saccharides. These two classes use the sugar as the alcohol, and a conserved Ser-His-Asp catalytic triad as the acid for the extraction of the acetyl residue from the polysaccharide chain. Chitin deacetylase (CE4, EC 3.5.1.41) operate by metal-assisted acid/base catalysis with an oxyanion intermediate state [17]. As with the chitosanases we noticed the deacetylases are moderately expanded in the mycoparasitic clades ST and HV (average:5) and reduced in the mainly saprotrophic clade SL (average:4). *T. atroviride* and *T. virens* (6 and 7, respectively) have the highest number within the selected genomes ([14] and this study). The N-acetylglucosamine-6-phosphate deacetylase (CE9, EC 3.5.1.25) is involved in GlcNAc catabolism in *Trichoderma* spp. [18] and a single enzyme is present in every investigated genome, with the only exception of *T. brevicompactum* and *T. gamsii,* which have a second copy in this gene family.

### Auxiliary Activity

Although 7 families of lytic polysaccharide monooxygenases are present in this category only 3 families of the Auxiliary Activities (AA) categories have been found to be involved in cell wall metabolism in *Trichoderma* spp. In total we identified 163 genes falling into these 3 families AA1, AA7, and AA11 in the *Trichoderma* genomes.

These redox enzymes act in conjunction with other CAZymes and are implicated in auxiliary activities to mobilize carbohydrates from recalcitrant material. The AA1-family laccases (EC 1.10.3.2), for example, are multicopper oxidases with a wide variety of complex substrates, such as lignin. And in *Cryptococcus neoformans* a laccase has been implicated as cell wall bound virulence factor, since it is involved in melanin production as reviewed in [19]. In *Trichoderma* spp. between 8 and 10 AA1 enzymes were identified. AA7 glucooligosaccharide-/ chitooligosaccharide oxidases (EC 1.1.3.-) and the AA11 copper-dependent lytic polysaccharide monooxygenases (EC1.14.99.53) are involved in oxidative cleavage of chitin chains and between 1 and 2.7 genes are present on average, respectively, in the analyzed *Trichoderma* genomes.

Pfam category

The thirteen analyzed Trichoderma genomes were further mined for the presence of the proposed protein Pfam categories for cell wall metabolism according to Muszewska et al., [2]. Interestingly, three Pfam-families that were found in human pathogenic fungi could not be identified in any of the *Trichoderma* spp. PF05390, PF10287 associated with glucan metabolism and PF12296 encoding a HsbA related surface binding protein A in *Aspergillus* spp. were not present in *Trichoderma* spp.

### PF00080 and PF00199

On average two genes corresponding to the PF00080/IPR001424 superoxide dismutase are present in *Trichoderma* spp. and are responsible for removing superoxide from the cell. These ubiquitous metalloproteins prevent damage by oxygen-mediated free radicals.

On average 7 PF00199/ IPR011614 catalase genes are present in *Trichoderma* spp. These are very common among all living aerobic organisms and serve as antioxidant enzymes that catalyze the conversion of hydrogen peroxide to water and molecular oxygen, serving to protect cells from its toxic effects and function in decomposition of hydrogen peroxide. Superoxide and hydrogen peroxide are normal by-product of aerobic respiration and produced by a number of reactions, including oxidative phosphorylation and photosynthesis. The dismutase and catalase enzymes have a very high catalytic efficiency and affectively prevent damage caused by the radicals.

### PF00399 and PF12454

Some, but not all *Trichoderma* spp. have one PF00399/ IPR000420 PIR protein, containing internal repeats of the highly conserved PIR-region of 18 to 19 residues. In yeast four highly homologous members of this family are located on the cell wall. A member of the PF12454 /Ecm33 family was identified in all *Trichoderma* spp. This GPI-anchored glycoprotein is an essential cell wall component and important for cell wall integrity.

# Phylogenetic analysis of the chitin deacetylases and chitosanases in the *Trichoderma* spp.

For the phylogenetic analysis of the chitin deacetylases and chitosanases a manual BLAST search was performed in the 14 *Trichoderma* species with the query sequences from all six identified enzymes from both families (CE4 and GH75) that had been investigated previously in *T. atroviride* [14].

The phylogenetic analysis using MEGA11 revealed that the CE4 chitin deacetylases can be grouped into six distinct groups. Note, that the identified four CDA3 orthologs, which in contrast to the other enzymes, have no signal peptide, are only present in the mycoparasitic ST-group and that the five identified CDA6 orthologs are also only present in members of the two mycoparasitic groups (ST or HV).

A similar picture is observed in the GH75 chitosanases. Again, the six enzymes group very well with their orthologs in the other species, but CHO2 and CHO6 orthologs are missing from the saprophytic group SL. Interestingly, CHO4, is only present in *T. atroviride*, and diverges quite distantly from the CHO1/CHO2 branch. The only distant ortholog of CHO4 could be identified in *T. hamatum* but does not directly group with CHO4. Interestingly, the phylogenetic analysis showed further that the average pairwise distance of the chitosanases is 0.38541 compared to chitin deacetylases, where it is 0.68201, revealing a stronger homology between the chitosanases than between the chitin deacetylases.

# References:

1. Kubicek CP, Steindorff AS, Chenthamara K, Manganiello G, Henrissat B, Zhang J, Cai F, Kopchinskiy AG, Kubicek EM, Kuo A: **Evolution and comparative genomics of the most common Trichoderma species**. *BMC genomics* 2019, **20**(1):1-24.

2. Muszewska A, Piłsyk S, Perlińska-Lenart U, Kruszewska JS: **Diversity of cell wall related proteins in human pathogenic fungi**. *Journal of Fungi* 2017, **4**(1):6.

3. Drula E, Garron M-L, Dogan S, Lombard V, Henrissat B, Terrapon N: **The carbohydrate-active enzyme database: functions and literature**. *Nucleic acids research* 2022, **50**(D1):D571-D577.

4. Mistry J, Chuguransky S, Williams L, Qureshi M, Salazar GA, Sonnhammer EL, Tosatto SC, Paladin L, Raj S, Richardson LJ: **Pfam: The protein families database in 2021**. *Nucleic acids research* 2021, **49**(D1):D412-D419.

5. Kubicek CP, Herrera-Estrella A, Seidl-Seiboth V, Martinez DA, Druzhinina IS, Thon M, Zeilinger S, Casas-Flores S, Horwitz BA, Mukherjee PK *et al*: **Comparative genome sequence analysis underscores mycoparasitism as the ancestral life style of Trichoderma**. *Genome biology* 2011, **12**(4):R40.

6. Baroncelli R, Zapparata A, Piaggeschi G, Sarrocco S, Vannacci G: **Draft whole-genome sequence of Trichoderma gamsii T6085, a promising biocontrol agent of Fusarium head blight on wheat**. *Genome announcements* 2016, **4**(1):e01747-01715.

7. Druzhinina IS, Chenthamara K, Zhang J, Atanasova L, Yang D, Miao Y, Rahimi MJ, Grujic M, Cai F, Pourmehdi S: **Massive lateral transfer of genes encoding plant cell wall-degrading enzymes to the mycoparasitic fungus Trichoderma from its plant-associated hosts**. *PLoS genetics* 2018, **14**(4):e1007322.

8. Studholme DJ, Harris B, Le Cocq K, Winsbury R, Perera V, Ryder L, Ward JL, Beale MH, Thornton CR, Grant M: **Investigating the beneficial traits of Trichoderma hamatum GD12 for sustainable agriculture—insights from genomics**. *Frontiers in plant science* 2013, **4**:258.

9. Fanelli F, Liuzzi VC, Logrieco AF, Altomare C: **Genomic characterization of Trichoderma atrobrunneum (T. harzianum species complex) ITEM 908: insight into the genetic endowment of a multi-target biocontrol strain**. *BMC genomics* 2018, **19**(1):1-18.

10. Proctor RH, McCormick SP, Kim H-S, Cardoza RE, Stanley AM, Lindo L, Kelly A, Brown DW, Lee T, Vaughan MM: **Evolution of structural diversity of trichothecenes, a family of toxins produced by plant pathogenic and entomopathogenic fungi**. *PLoS pathogens* 2018, **14**(4):e1006946.

11. Martinez D, Berka RM, Henrissat B, Saloheimo M, Arvas M, Baker SE, Chapman J, Chertkov O, Coutinho PM, Cullen D: **Genome sequencing and analysis of the biomass-degrading fungus Trichoderma reesei (syn. Hypocrea jecorina)**. *Nature biotechnology* 2008, **26**(5):553-560.

12. Yang D, Pomraning K, Kopchinskiy A, Karimi Aghcheh R, Atanasova L, Chenthamara K, Baker SE, Zhang R, Shen Q, Freitag M *et al*: **Genome sequence and annotation of Trichoderma parareesei, the ancestor of the cellulase producer Trichoderma reesei**. *Genome Announcements* 2015, **3**(4):e00885-00815.

13. Schwerdt J, Qiu H, Shirley N, Little A, Bulone V: **Phylogenomic analyses of nucleotide-sugar biosynthetic and interconverting enzymes illuminate cell wall composition in fungi**. *mBio* 2021, **12**(2):e03540-03520.

14. Kappel L, Münsterkötter M, Sipos G, Escobar Rodriguez C, Gruber S: **Chitin and chitosan remodeling defines vegetative development and Trichoderma biocontrol**. *PLoS Pathog* 2020, **16**(2):e1008320.

15. Mélida H, Sain D, Stajich JE, Bulone V: **Deciphering the uniqueness of Mucoromycotina cell walls by combining biochemical and phylogenomic approaches**. *Environmental Microbiology* 2015, **17**(5):1649-1662.

16. Seidl V, Huemer B, Seiboth B, Kubicek CP: **A complete survey of *Trichoderma* chitinases reveals three distinct subgroups of family 18 chitinases**. *FEBS J* 2005, **272**(22):5923-5939.

17. Grifoll-Romero L, Pascual S, Aragunde H, Biarnés X, Planas A: **Chitin deacetylases: Structures, specificities, and biotech applications**. *Polymers* 2018, **10**(4):352.

18. Kappel L, Gaderer R, Flipphi M, Seidl‐Seiboth V: **The N‐acetylglucosamine catabolic gene cluster in Trichoderma reesei is controlled by the Ndt80‐like transcription factor RON1**. *Molecular microbiology* 2016, **99**(4):640-657.

19. Zhu X, Williamson PR: **Role of laccase in the biology and virulence of Cryptococcus neoformans**. *FEMS yeast research* 2004, **5**(1):1-10.
