# Supplementary material for: A comparative cell wall analysis of Trichoderma spp. confirms a conserved polysaccharide scaffold and suggests an important role for chitosan in mycoparasitism
Source: Microbiol Spectr. 2024 Jun 25;12(8):e03495-23. doi: 10.1128/spectrum.03495-23 (PMC11302013; doi:10.1128/spectrum.03495-23)
Supplement: Supplemental material — Supplemental figure legends. [file spectrum.03495-23-s0007.docx]

# Supplementary figure legends

**FIG S1.** Gel electrophoreses of PCR products generated from clones of the *T. atroviride fks1* deletion mutant, were the whole reading frame of *fks1* was replaced with a hygromycin B cassette. Lane1: 1kb ladder; lane 2: genetically instable *fks1* deletion mutant; lane 3: *fks1* deletion mutant; lane 4: reverted *fks1* deletion mutant. * indicates full length *fks1* gene at 5,772 bp with ca. 3000 bp flanking region; arrow indicates integrated hygromycin B phosphotransferase marker cassette.

**FIG S2.** Determination of β-1,3-1,4-glucan in percent (%) of the total cell wall dry weight from mycelium of *T. atroviride* (black bars) and *T. reesei* (grey bars) using the β-Glucan Assay Kit for Mixed Linkage from Megazyme. Values are means ± SD of two biological with two technical determinations as described in Materials and Methods.

**FIG S3.** SDS-PAGE showing the crude lysate (CL), the microsomal fraction (MF), the detergent extracted membrane proteins (DE) and the precipitated residue (RD) from the detergent extraction.

**FIG S4.** Harvesting areas for determination of nascent chitosan in cell walls of *T. atroviride* in confrontation with the host *S. sclerotiorum* (condition: before contact BC) or condition: alone.

**FIG S5.** A. Overgrowth *of Sclerotinia sclerotiorum* (Ss) by *T. atroviride* IMI (Ta) in a confrontation assay on PDA, three (left) and 10 days (right) after inoculation. B. Hyphal attachment of *T. atroviride* on host hyphae (*S. sclerotiorum*) during mycoparasitic attack.
